# Anti-Wear Property of Aluminum–Silicon Alloy Treated by Chemical Etching, Mechanical Honing and Laser Finishing

**DOI:** 10.3390/ma12081273

**Published:** 2019-04-18

**Authors:** Fengming Du, Chengdi Li, Zetian Mi, Yan Shen, Ruoxuan Huang, Xiaoguang Han, Yong Dong, Jiujun Xu

**Affiliations:** 1 College of Marine Engineering, Dalian Maritime University, Dalian 116026, China; fengming_du@163.com (F.D.); marinedmu@163.com (Y.S.); 2 Xinyu Key Laboratory of Materials Technology and Application for Intelligent Manufacturing, Xinyu University, Xinyu 338004, China; lichengdi17@163.com; 3 College of Information Science and Technology, Dalian Maritime University, Dalian 116026, China; 4 Key Lab of Ship-Maintenance & Manufacture, Dalian Maritime University, Dalian 116026, China; rxhuang@dlmu.edu.cn (R.H.); xghandl@163.com (X.H.); xujiujun@163.com (J.X.); 5 School of Materials and Energy, Guangdong University of Technology, Guangzhou 510006, China

**Keywords:** anti-wear, aluminum–silicon alloy cylinder liner, chemical etching, mechanical honing, laser finishing

## Abstract

To enhance the anti-wear property of aluminum–silicon (Al–Si) alloy, three processing technologies—chemical etching, mechanical honing and laser finishing—were compared in terms of their effects on anti-wear performance. The treated Al–Si alloy cylinder liner samples were worn against a piston ring by a reciprocating sliding tribotester; the anti-wear performance was represented by the friction coefficient and wear loss; and the wear mechanism was determined by establishing stress contact models. The results showed that the best time for both the chemical etching and mechanical honing treatments was 2 min, and the optimal laser power was 1000 W for the laser finishing treatment. The three processing technologies could all remove the aluminum layer and make the silicon protrude on the surface to avoid the plastic flow of aluminum during the friction process. The laser finishing could not only protrude the silicon particle but also make its edge rounded and smooth, which decreased the stress concentration. Therefore, the Al–Si alloy cylinder liner treated with laser finishing had the best anti-wear performance.

## 1. Introduction

Lightweight materials in automobile construction help save energy. Aluminum–silicon (Al–Si) alloy has been widely used in lightweight engine components for building fuel-efficient vehicles [1,2,3,4,5,6]. 

When an Al–Si alloy rubs against other metals, the plastic flow of aluminum may easily occur on the surface and be prone to adhesive wear, resulting in a bad anti-wear property [7,8,9,10,11]. The Al–Si alloy cylinder liner material has the characteristics of a soft aluminum matrix inlaid with hard silicon particles, and the ideal surface is one in which the hard silicon particles are convex to bear the heavy load, and the relatively concave aluminum base portion can store oil to promote lubrication. Such a structure can enhance the anti-wear properties of Al–Si alloy cylinder liners [12,13,14].

At present, the surface of the standard Al–Si alloy cylinder liner is usually treated by chemical etching, so that the surface aluminum is etched and then the silicon particles are exposed from the surface to bear load, leading to a better tribological property [15,16,17]. However, chemical etching pollutes the environment. Traditional honing technology is widely used for the cast iron cylinder liner [18,19,20], it is by means of the oilstone embedded in the honing head to process the surface, but it may not be suitable for an Al–Si alloy cylinder liner because the aluminum may easily cover the silicon during the traditional honing process. Laser processing technology is also widely utilized in manufacturing to improve the properties of materials, such as laser cladding, laser surface alloying and laser remelting [21,22,23,24].

In this paper, in order to improve the anti-wear property of Al–Si alloy cylinder liners, a new self-designed honing machine was developed for the honing processing, which was different from the traditional honing machine; the new honing method could be innovative and suitable for an Al–Si alloy cylinder liner. A CO_2_ continuous transverse-flow laser was used for laser finishing, so that the two processing technologies could be compared with the traditional chemical etching. The optimized parameters for chemical etching, mechanical honing and laser processing were researched to obtain the best anti-wear performance. Additionally, stress models were established to calculate the contact stress; the influence mechanisms of three different processing techniques on the wear properties of the cylinder liner surface were also discussed in details.

## 2. Experimental Process

### 2.1. Materials

**Figure 1:**
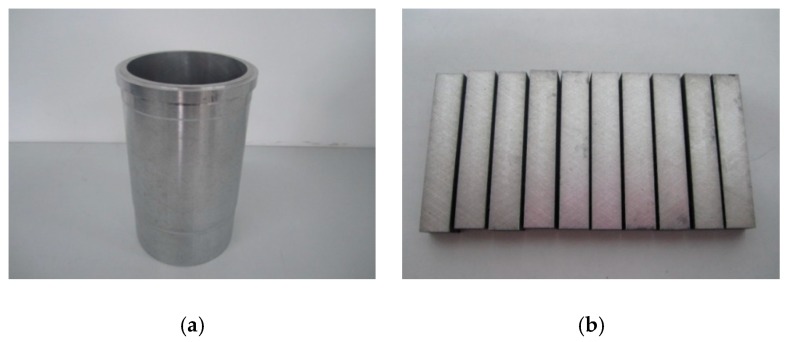
Al–Si alloy cylinder liner and samples. (**a**) Cylinder liner; (**b**) cylinder liner samples.

**Figure 2:**
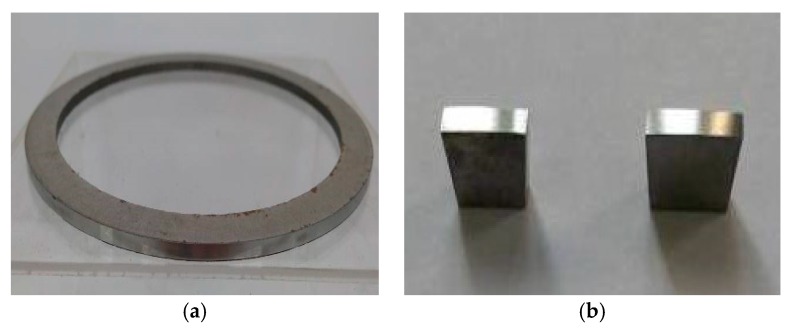
Piston ring and samples. (**a**) Piston ring; (**b**) piston ring samples.

The compositions of the Al–Si alloy cylinder liner are shown in Table 1. The diameter of the Al–Si alloy cylinder liner was 110 mm and the thickness was 8 mm, it was equally divided into 40 samples in the circumferential direction, and the length of each sample was 9 mm, as shown in Figure 1. A CKS (Chrom–Keramik–Schicht) piston ring was selected to wear against the cylinder liner. The CKS piston ring was achieved by chromium-based ceramic composite plating; the chromium layer was about 50 μm, and Al_2_O_3_ ceramic particles were implanted in the micro crack of the chromium layer. In order to ensure experimental repeatability, the piston ring was customized and had no ring gap. The piston ring’s diameter was 110 mm and height was 3 mm, it was divided into 32 samples in the circumferential direction, as shown in Figure 2. The roughness of the Al–Si alloy cylinder liner and CKS piston ring were 0.89 μm Ra and 0.24 μm Ra, respectively.

### 2.2. Chemical Etching

**Figure 3:**
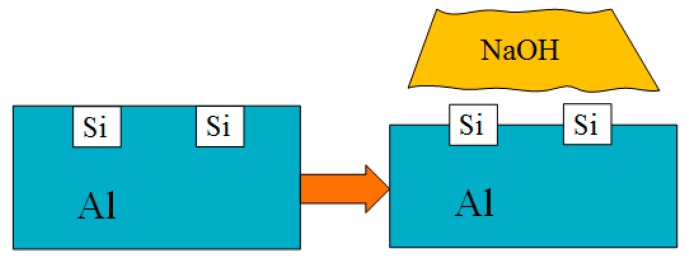
Chemical etching process of the Al–Si alloy cylinder liner.

The Al–Si alloy cylinder liner samples were etched by a five percent NaOH solution at 20 °C for different times, with these etching times being set to 1 min, 2 min, 2.5 min, 3 min and 3.5 min. The samples were immersed in the NaOH solution and were quickly taken out after the set time was reached and flushed with running water. Then they were placed in gasoline and alcohol for ultrasonic cleaning. Chemical etching can etch the aluminum and expose the silicon particles, as shown in Figure 3.

### 2.3. Mechanical Honing

**Figure 4:**
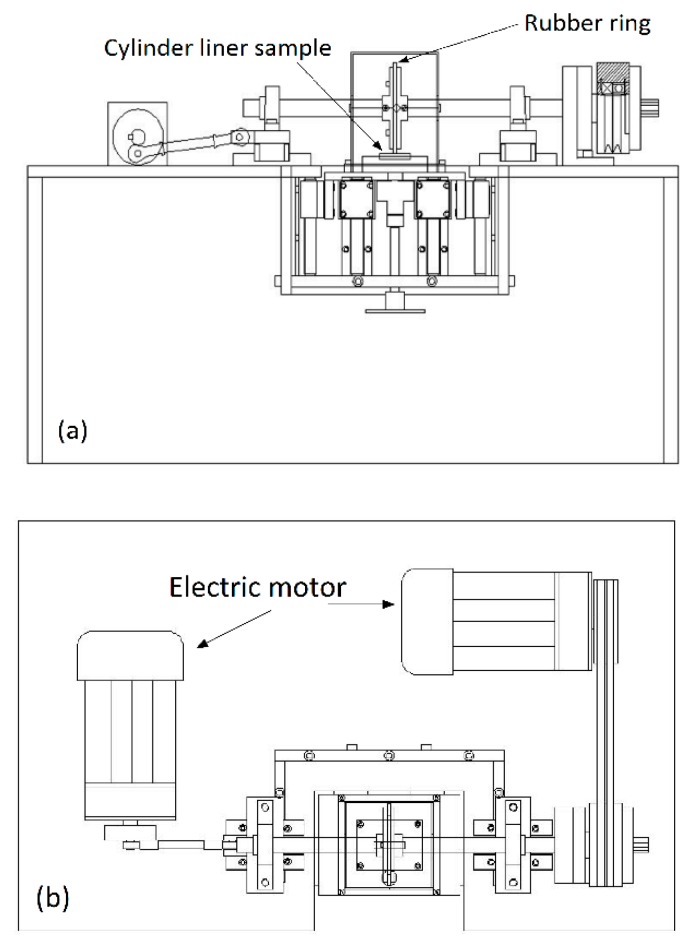
Diagram of mechanical honing equipment. (**a**) Front view; (**b**) vertical view.

**Figure 5:**
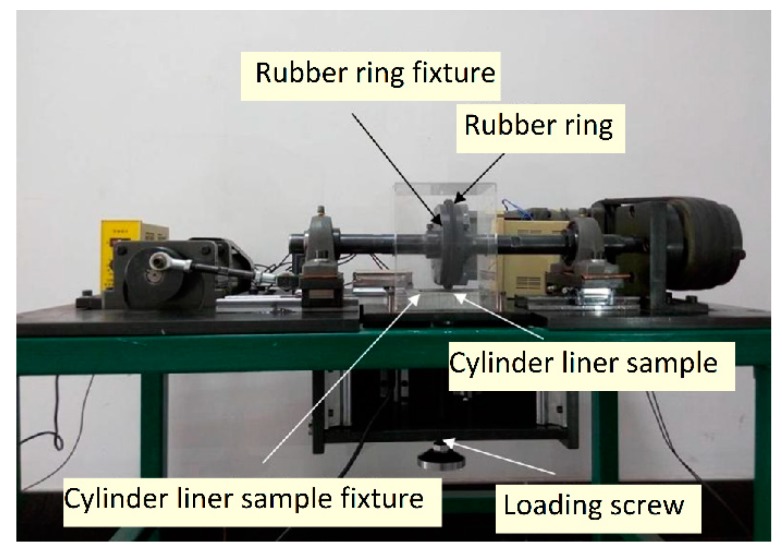
Mechanical honing equipment.

**Figure 6:**
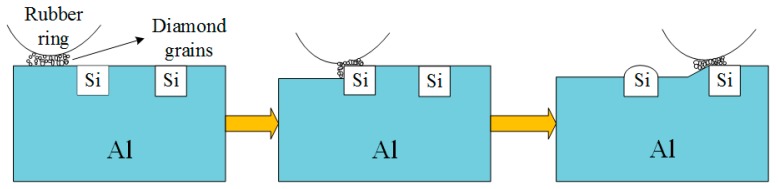
Mechanical honing process of the Al–Si alloy cylinder liner.

In order to achieve the purposes of micro-chamfering the corners of the protruding silicon particles by mechanical means, and strictly planning the technical parameters with the precise control of the equipment, a new type of mechanical honing equipment was developed, as shown in Figure 4 and Figure 5. The self-designed honing machine was able to perform both rotary and linear reciprocating motions and had the advantages of controllable parameters, simple operation, high processing efficiency and low cost. It was specially designed for the surface characteristics of the Al–Si cylinder liners. Figure 6 shows the process of the mechanical honing of the Al–Si alloy cylinder liner samples. The rubber had good elasticity, and the diamond particles were particles with a higher hardness. It can be seen that when the rubber ring ground the surface of the cylinder liner with diamond particles, the aluminum on the surface was worn away. When grinding the silicon particles, only the corners of the silicon were worn away, because of the significant hardness of the silicon particles; therefore, the mechanical honing processing caused the silicon particles to protrude from the surface, and the corners were rounded. There existed some burrs on the surface due to the machining marks.

### 2.4. Laser Finishing

A CO_2_ continuous transverse-flow laser was used for the laser finishing of the Al–Si alloy cylinder liner samples. The advantage of the continuous transverse-flow laser was that the laser beam was continuous and the energy was stable.

**Figure 7:**
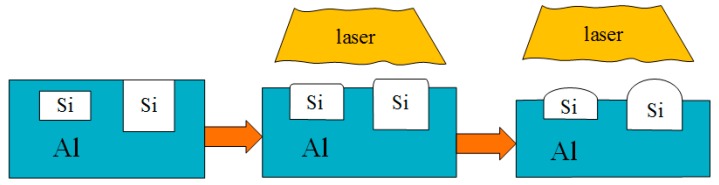
Laser finishing process of the Al–Si alloy cylinder liner.

Figure 7 is a schematic diagram of the principle of laser finishing. The laser could raise the surface temperature of the cylinder liner to a high temperature in a short time. The aluminum on the surface began to melt due to the high temperature, while the silicon with a high melting point melted later, leading to that silicon being exposed on the surface. According to the lowest surface energy principle, the silicon particles melted from the convex corners and became rounded by the surface tension. The laser power affected the melt of the aluminum and silicon, and in turn affects the protrusion height of the silicon particles.

### 2.5. Tribotests

**Figure 8:**
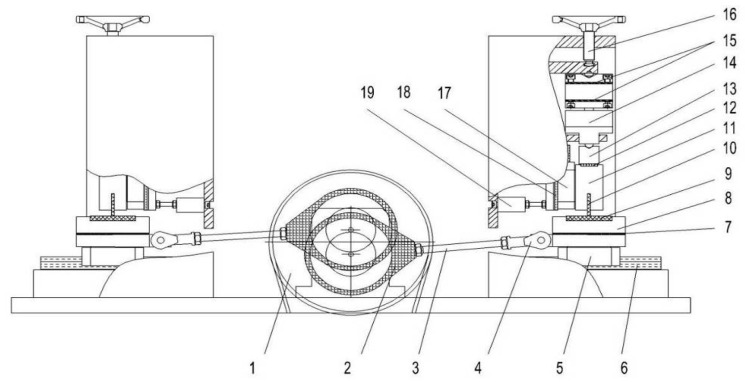
The geometry of the tribotester: 1—Belt pulley, 2—Eccentric shaft, 3—Connecting rod, 4—Knuckle bearing, 5—Sliding block, 6—Guide rail, 7—Thermal baffle, 8—Heater, 9—Al–Si alloy cylinder liner sample, 10—CKS piston ring sample, 11—Pressure head, 12—Roller pin, 13—Self-aligning slider, 14—Pressure sensor, 15—Plate spring, 16—Knob screw, 17—Sliding block, 18—Guide rail, 19—Friction force sensor.

Tribotests were conducted on a reciprocating sliding tribotester [25], as shown in Figure 8. The tribotester could adjust speeds (5 r/min–500 r/min), contact pressure (5 MPa–380 MPa), and temperatures (30–300 °C) between the piston ring and the cylinder liner. In this experiment, the sliding speed and the temperature were set to 200 r/min and 150 °C, respectively. The contact pressure consisted of two stages, which were running-in stage and steady stage; the first stage was a low contact pressure of 5 MPa for 1 h to reduce the effect of burrs on test repeatability and the second stage was a high contact pressure of 20 MPa for 4 h. The lubricating oil was RP-4652D (15W-40/CF-4) which contained Zinc Dialkyl Dithiophosphates (ZDDP) additives, and it was supplied at a speed of 0.1 mL/min. The general physical and chemical properties of RP-4652D were listed in Table 2. In this test, there were 5 types of samples (according to different etching times) for chemical etching, 4 types of samples for mechanical honing and 5 types of samples for laser finishing, with each type of sample being repeated four times to obtain stable results. 

Wear performance was presented by friction coefficient and wear loss. The friction coefficient was the ratio of friction to normal contact pressure, and it was obtained during the steady state. Wear loss was measured by the difference in weight before and after the tribotest. The surface morphologies were analyzed by laser scanning confocal microscopy (OLYMPUS LEXT OLS4000, Tokyo, Japan) and a scanning electron microscope (PHILIPS-30TMP, Amsterdam, Netherlands).

## 3. Experimental Results

### 3.1. Surface Morphology of the Cylinder Liner

**Figure 9:**
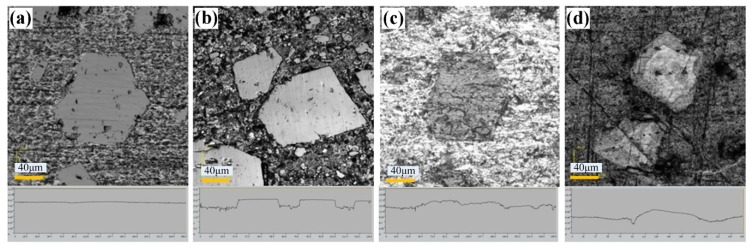
Polished surface morphologies and outlines of the cylinder liner: (**a**) untreated; (**b**) chemical etching; (**c**) mechanical honing; (**d**) laser finishing.

**Figure 10:**
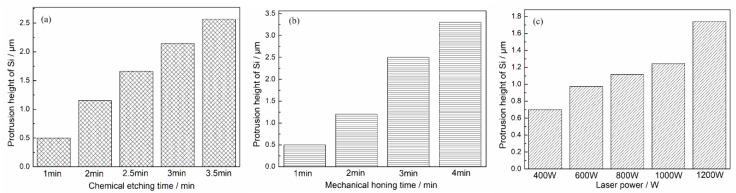
Protrusion height of silicon particle after different treatment: (**a**) Chemical etching; (**b**) mechanical honing; (**c**) laser finishing.

Figure 9 shows the surface morphologies of original and treated Al–Si alloy cylinder liners. As seen in Figure 9a, the surface of the untreated cylinder liner was flat, and aluminum and silicon were on the same plane. Chemical etching may scour the aluminum substrate on the surface, exposing the silicon particles, as shown in Figure 9b. The outline of the silicon after mechanical honing can be seen as a convex surface, and the corners were rounded, but there were some burrs, as shown in Figure 9c. After laser finishing, the silicon particles were exposed on the surface, and the edges of the silicon particles were rounded and smooth, as shown in Figure 9d. Apparently, the three processing technologies could all make the silicon particles protrude on the surface; as with the increase in chemical etching time, mechanical honing time and laser power, the protrusion height of silicon particles also increased, as shown in Figure 10.

### 3.2. Anti-Wear Performance after Chemical Etching

**Figure 11:**
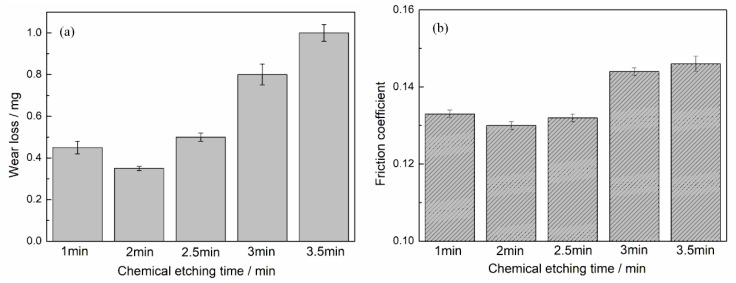
Effect of chemical etching time on the (**a**) wear loss and (**b**) friction coefficient.

Wear tests of cylinder liner samples that had been chemically etched at different times were respectively conducted on the tribotester. The protrusion height of silicon particles was different according to the different etching times, which would result in differences in wear performance. As the chemical etching time increased, the friction coefficient and wear loss tended to first decrease and then increase, as shown in Figure 11. If the time was short, the chemical etching did not remove sufficient aluminum from the surface to expose the silicon. If the time was long, the protrusion height of silicon was high, and the silicon may peel off easily and become abrasive grains after wear. When the chemical etching time was 2 min, the wear loss and friction coefficient both reached the minimum value.

### 3.3. Anti-Wear Performance after Mechanical Honing

**Figure 12:**
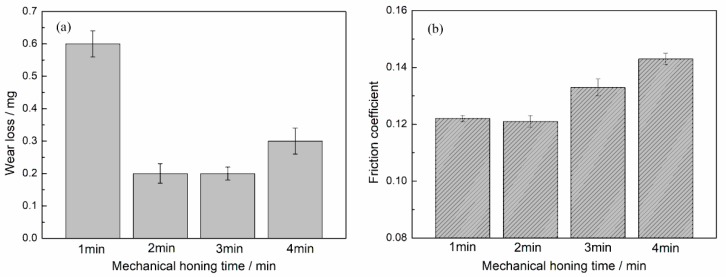
Effect of mechanical honing time on the (**a**) wear loss and (**b**) friction coefficient.

Figure 12 shows the wear loss and friction coefficient at different mechanical honing times. The minimum wear loss was observed at the time of 2 min or 3 min; the friction coefficient first increased and then decreased with the increase in mechanical honing time. When the mechanical honing time was 2 min, both the wear loss and friction coefficient reached a minimum value.

### 3.4. Anti-Wear Performance after Laser Finishing

**Figure 13:**
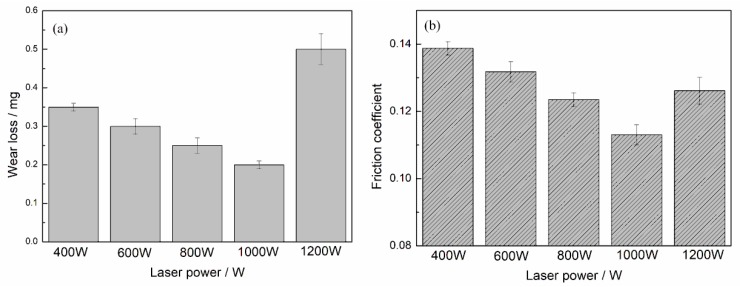
Effect of laser power on the (**a**) wear loss and (**b**) friction coefficient.

The cylinder liner samples that had been laser finished at different laser power were also tested on the tribotester. The distributions of the friction coefficient and wear loss demonstrated the same rule: if the laser power was too small or too large, it did not achieve good results. The smallest wear loss was observed at a laser power of 1000 W, and the friction coefficient showed the same tendency, as shown in Figure 13.

### 3.5. Anti-Wear Performance Comparisons

The optimal Al–Si alloy cylinder liners after the three surface treatment methods were chosen to compare the wear performance, and the protruding heights of the silicon particles were approximately 1.2 μm for all samples.

**Figure 14:**
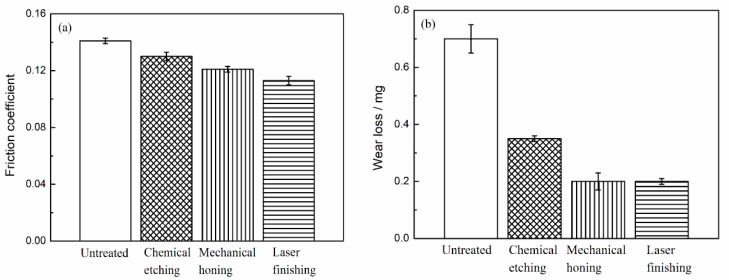
Friction coefficient (**a**) and wear loss (**b**).

Figure 14 shows the comparisons of the friction coefficient and wear loss of Al–Si alloy cylinder liners with different surface treatments. It can be seen that when the piston ring was worn against the untreated cylinder, the friction coefficient was approximately 0.14, which was the largest coefficient. The friction coefficients of samples treated by the three surface processing methods were lower than those of the untreated samples, because the protrusion of the silicon particles reduced the adhesive wear and promoted lubrication. Among them, the laser finishing cylinder liner had the smallest friction coefficient: 0.11. Because the silicon particles were rounded and smooth, the stress concentration and friction were reduced, and so the friction coefficient was also reduced. It can be seen that the wear loss of the untreated cylinder liner was approximately 0.7 mg, the wear loss of the chemical etching was approximately 0.35 mg, and the wear loss of the mechanical honing was almost the same as that of the laser finishing, which was approximately 0.2 mg.

### 3.6. Surface Morphology of the Worn Cylinder Liner

**Figure 15:**
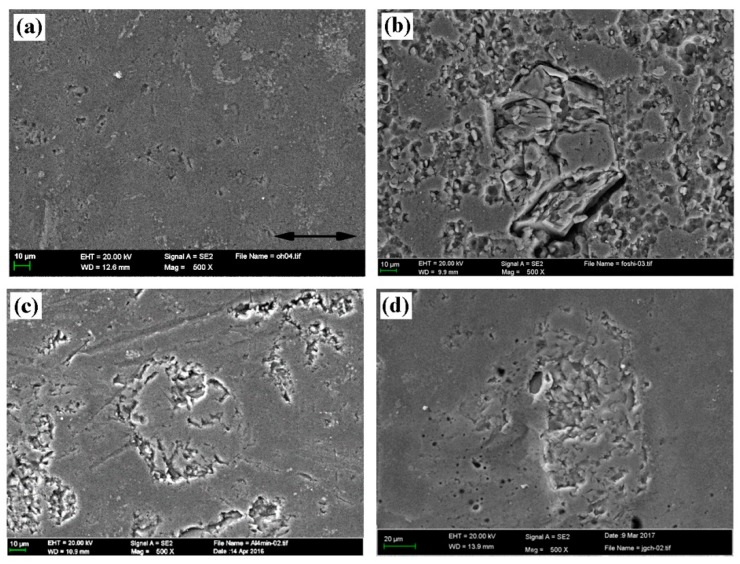
The worn surface morphologies of the Al–Si alloy cylinder liner: (**a**) Untreated; (**b**) chemical etching; (**c**) mechanical honing; (**d**) laser finishing.

Figure 15 shows the worn surface topography of the Al–Si alloy cylinder liner after a tribotest. Figure 15a shows the surface of the untreated cylinder liner; it was smooth, and no obvious protruding silicon particles were observed. In the friction process, the surface aluminum layer was in contact with the piston ring and was rolled over the surface during the reciprocating motion. Figure 15b shows the surface of the chemically etched cylinder liner; it was worn and damaged at the corners, the convex silicon particles were sharp, and the stress was concentrated during the friction process, which broke easily at the corners. Figure 15c shows the surface of the mechanically honed cylinder liner; during the friction process, the processing burrs were significantly worn away, and the silicon particles were not damaged. Figure 15d shows the surface of the laser finished cylinder liner; the silicon particles remained on the surface, the bonding state with the aluminum substrate remained robust, and the wear was slight.

## 4. Simulation

**Figure 16:**
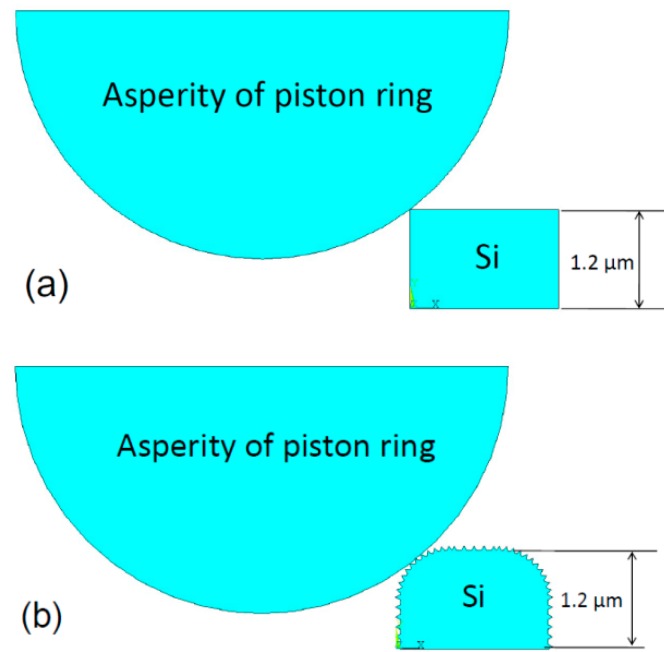

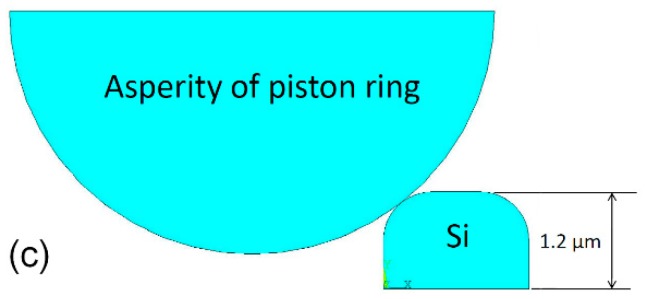
Geometric representation of the contact model: (**a**) Chemical etching model; (**b**) mechanical honing model; (**c**) laser finishing model.

The wear models of the piston ring worn against the chemically etched cylinder liner, the mechanically honed cylinder liner, and the laser finished cylinder liner were established to compare the contact stress of the silicon particles in a static state, as shown in Figure 16. The material property parameters are shown in Table 3.

The assumptions of the stress model were made as follows:

1. The asperity of the piston ring only contacted with the silicon particles.
2. The piston ring/silicon particle contact was rigid to flexible, so the piston ring elements could not invade the silicon.
3. The worn depth in the three cases was the same, so a downward displacement could be applied on the piston ring, and the value displacement value was set to 0.05 μm.

The boundary conditions for the stress model were: 

1. The bottom nodes of the silicon particle were fixed:

*U_x_* = *U_y_* = 0 (1)

where *U_x_* and *U_y_* were the degrees of freedom along the x- and y-direction.

2. A downward displacement was set to the piston ring:

*U_y_* = −0.05 μm (2)

**Figure 17:**
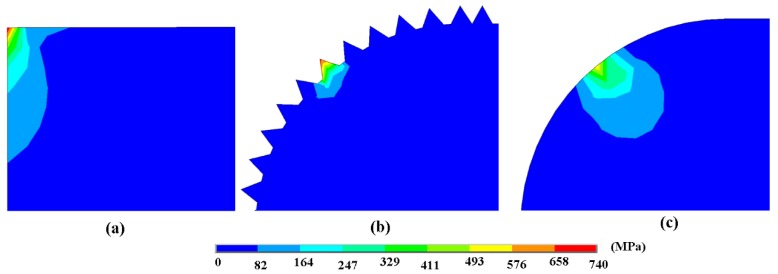
Contact stress distribution of silicon particles at the contact areas: (**a**) Chemical etching model; (**b**) mechanical honing model; (**c**) laser finishing model.

Figure 17 shows the contact stress of the silicon particles under three different process conditions. After chemical etching, the maximum stress occurred at the corners of the silicon particles with a value of 737 MPa. While the edge of the silicon particles after laser finishing was relatively smooth, and the maximum stress was 561 MPa, which reduced the stress concentration. After mechanical honing, there existed many burrs and the maximum stress of the burr was 695 MPa, which may make the burrs peel off easily. When the burrs were smoothed during the friction process, the surface topography after mechanical honing would be similar to the ones after laser finishing. It can be seen that during the contact process, the silicon particles on the surface of the cylinder liner after chemical corrosion were subjected to greater stress, so that wear was more likely to occur, and the wear loss of the cylinder liner was the largest.

## 5. Mechanism of Wear Reduction

**Figure 18:**
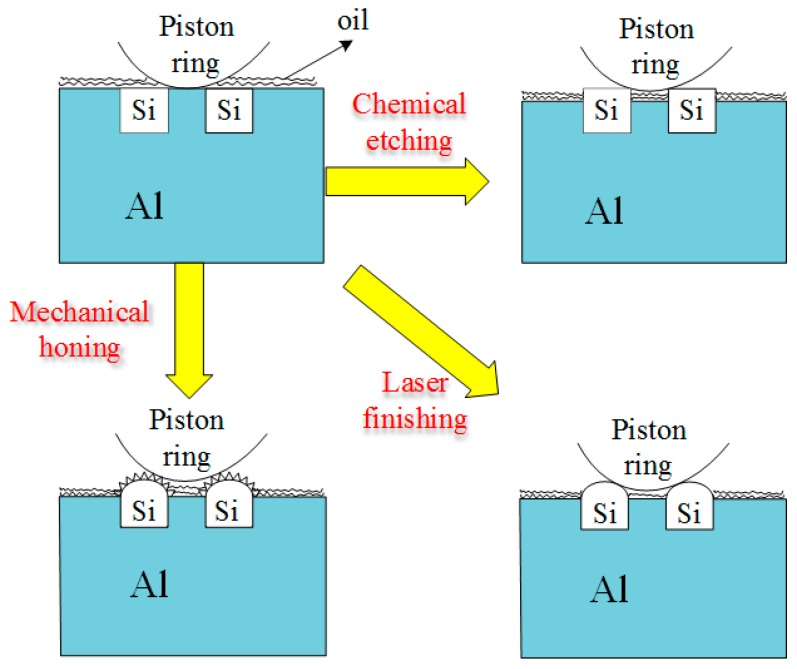
Schematic diagram of the wear mechanism.

Figure 18 shows the mechanism of wear reduction after different surface processing technologies. In the untreated cylinder liner, the silicon particles were buried in the aluminum matrix, and the aluminum matrix was in direct contact with the piston ring and prone to adhesive wear, resulting in a higher friction coefficient and wear loss. For all three methods of processing, the silicon particles on the surface of the liner were convex and could withstand heavy loads, and the recessed aluminum matrix could store oil and enhance lubrication under the boundary lubrication state. The chemically etched cylinder liner had sharp edged silicon particles. After mechanical honing, the silicon particles were rounded to reduce the stress concentration, but there were more burrs. After laser finishing, the silicon particles exhibited smooth rounded corners and reduced the stress concentration.

## 6. Conclusions

In this study, a new self-designed honing machine was developed, which was innovative and able to enhance the anti-wear property of an Al–Si alloy cylinder liner. Chemical etching, mechanical honing and laser finishing processing technologies were applied to an Al–Si alloy cylinder liner, and the anti-wear performance was tested by a reciprocating tribotest, the optimized parameters for chemical etching, mechanical honing and laser processing were obtained through the research. The following conclusions may be drawn:

1. When etched by a five percent NaOH solution, the friction coefficient and wear loss of the Al–Si alloy cylinder liner tended to first decrease and then increase with the increase in the etching time; the optimal etching time was found to be 2 min.
2. Based on the self-designed honing machine, the mechanical honing time should not be too long or too short for the Al–Si alloy cylinder liner; the optimal value was found to be 2 min.
3. The friction coefficient and wear loss of the Al–Si alloy cylinder liner also first decreased and then increased with the increase of laser power; the optimum laser power was found to be 1000 W.
4. Chemical etching, mechanical honing, and laser finishing were all found to remove the surface aluminum layer and result in the protrusion of the silicon particles, which bear the load; meanwhile, the lubricating oil was retained in the concave surface to enhance lubrication, resulting in good anti-wear performance.
5. Laser finishing resulted in rounded and smooth edges on the silicon particles, which showed the best anti-wear property during the three processing technologies.

## Figures and Tables

**Table 1:** The chemical compositions of the Al–Si alloy cylinder liner.

Element	Al	Si	Fe	Cu	Mg	Zn
wt.%	71	20.1	0.9	5	0.6	1.0

**Table 2:** General physical and chemical property of RP-4652D.

Item	Value	Test Method
Kinematic viscosity 100 °C (mm^2^·s^−1^)	14.75	GB/T 265
Cryogenic dynamic viscosity −25 °C (MPa·s)	5480	GB/T 6538
Cryogenic pumping viscosity (no yield stress) −30 °C (MPa·s)	240	SH/T 0562
Pour point (°C)	−36	GB/T3535
Flash point (open) (°C)	230	GB/T3536
Moisture (wt.%)	<0.03	GB/T 260
Mechanical impurities (wt.%)	<0.01	GB/T 511
High temperature and high shear rate viscosity (150 °C, 106 s^−1^) (MPa·s)	4.0	SH/T 0618

**Table 3:** Material property parameters.

Material	Elasticity Modulus (GPa)	Poisson’s Ratio	Density (g/cm^3^)
Si	190	0.28	2.33
Al	71.7	0.33	2.7
